# Mechanisms of FUS1/TUSC2 deficiency in mesothelioma and its tumorigenic transcriptional effects

**DOI:** 10.1186/1476-4598-8-91

**Published:** 2009-10-24

**Authors:** Alla V Ivanova, Sergey V Ivanov, Ljudmila Prudkin, Daisuke Nonaka, Zhandong Liu, Anne Tsao, Ignacio Wistuba, Jack Roth, Harvey I Pass

**Affiliations:** 1 Hematology/Oncology Division, Vanderbilt Medical Center, Nashville, TN, USA; 2 Department of Cardiothoracic Surgery, NYU Langone Medical Center, New York, NY, USA; 3 Department of Thoracic/Head and Neck Medical Oncology, University of Texas M D Anderson Cancer Center, 1515 Holcombe Blvd, Unit 432, Houston, TX, USA; 4 Genomics and Computational Biology, University of Pennsylvania School of Medicine, Philadelphia, PA, USA; 5 Department of Thoracic and Cardiovascular Surgery, University of Texas MD Anderson Cancer Center, Houston, TX, USA

## Abstract

**Background:**

FUS1/TUSC2 is a novel tumor suppressor located in the critical 3p21.3 chromosomal region frequently deleted in multiple cancers. We previously showed that Tusc2-deficient mice display a complex immuno-inflammatory phenotype with a predisposition to cancer. The goal of this study was to analyze possible involvement of TUSC2 in malignant pleural mesothelioma (MPM) - an aggressive inflammatory cancer associated with exposure to asbestos.

**Methods:**

TUSC2 insufficiency in clinical specimens of MPM was assessed via RT-PCR (mRNA level), Representational Oligonucleotide Microarray Analysis (DNA level), and immunohistochemical evaluation (protein level). A possible link between TUSC2 expression and exposure to asbestos was studied using asbestos-treated mesothelial cells and ROS (reactive oxygen species) scavengers. Transcripional effects of TUSC2 in MPM were assessed through expression array analysis of TUSC2-transfected MPM cells.

**Results:**

Expression of TUSC2 was downregulated in ~84% of MM specimens while loss of TUSC2-containing 3p21.3 region observed in ~36% of MPMs including stage 1 tumors. Exposure to asbestos led to a transcriptional suppression of TUSC2, which we found to be ROS-dependent. Expression array studies showed that TUSC2 activates transcription of multiple genes with tumor suppressor properties and down-regulates pro-tumorigenic genes, thus supporting its role as a tumor suppressor. In agreement with our knockout model, TUSC2 up-regulated IL-15 and also modulated more than 40 other genes (~20% of total TUSC2-affected genes) associated with immune system. Among these genes, we identified CD24 and CD274, key immunoreceptors that regulate immunogenic T and B cells and play important roles in systemic autoimmune diseases. Finally, clinical significance of TUSC2 transcriptional effects was validated on the expression array data produced previously on clinical specimens of MPM. In this analysis, 42 TUSC2 targets proved to be concordantly modulated in MM serving as disease discriminators.

**Conclusion:**

Our data support immuno-therapeutic potential of TUSC2, define its targets, and underscore its importance as a transcriptional stimulator of anti-tumorigenic pathways.

## Background

Malignant Mesothelioma (MPM) is one of the most aggressive and devastating tumors, with a median survival of 10-12 months. MPM is a highly proliferative and locally invasive cancer that rapidly engulfs the pleural cavity, destroys normal lung structure, and results in cardiopulmonary compromise and progressive pain [1]. It is estimated that more than 90% of MPM cases are linked with exposure to asbestos [2]. Lodged asbestos fibers irritate mesothelial or pulmonary tissue causing chronic inflammation, and in the thorax, this persistent inflammation may eventually result in MPM or lung cancer [3]. Treatment of MPM with conventional therapies has proven to be largely unsuccessful [4]. Being an intrinsically drug resistant tumor, MPM shows only partial response to a combination of pemetrexed (Alimta) and cisplatin in ~41% of patients [5]. Animal studies, however, have demonstrated that MPM may respond to immunotherapy [6-8]. Thus, there is an urgent need to develop new effective therapies based on the knowledge of the key immunoregulatory molecules that could potentially suppress MPM growth.

Tumor suppressor genes (TSGs) play a major role in the pathogenesis of human cancers. Recurrent loss of heterozygosity in the 3p21.3 chromosomal region, as well as homozygous deletions and epigenetic inactivation of this area are observed in early stages of lung, breast, ovarian, liver, cervical, and other cancers suggesting a critical role for one or more genes that reside in 3p21.3. The products of these genes may act as "gatekeepers" in the molecular pathogenesis of common epithelial cancers [9-14].

FUS1 (TUSC2) was first characterized by us as a gene located in the minimal ~120 kb common area of loss in 3p21.3. The TUSC2 gene product is a small, basic, soluble, globular, and highly conserved protein, which is expressed in a variety of cell types. We have demonstrated that the TUSC2 protein resides mostly in mitochondria [15]. The high expression of Tusc2 during mouse embryogenesis and, specifically, in mouse embryonic stem cells (ESC)  suggests that TUSC2 has the potential as a cancer-causing gene. The tumor suppressor property of TUSC2 was confirmed experimentally [16-19] on lung cancer cell lines *in vitro *and on xenograft mice *in vivo*. Intratumoral and intravenous injections of TUSC2 into mice bearing xenografted A549 lung cancer resulted in significant inhibition of tumor growth and metastases. The biological function of the TUSC2 protein, however, is unknown and little data exist on the TUSC2-dependent signal transduction pathways. Most recently, TUSC2 was associated with the c-Abl/p53 pathway [20,21]. It has also been demonstrated that exogenous expression of TUSC2 using a nanoparticle-mediated gene transfer technique sensitizes NSCLC cells to cisplatin, resulting in a 4- to 6-fold increase in the TUSC2 tumor suppressor activity. The effect of TUSC2 on chemosensitivity was associated with down-regulation of MDM2, accumulation of p53, and activation of the Apaf-1 apoptotic pathway [22].

We have recently demonstrated that genetically modified mice lacking one or both copies of the Tusc2 gene develop an autoimmune disorder with an inflammatory background, and produce tumors at the sites of chronic inflammation [15]. These findings prompted us to investigate possible involvement of TUSC2 in human inflammatory cancers. Since MPM is one of the malignancies clearly linked to chronic inflammation induced by asbestos, we asked whether TUSC2 deficiency could be observed in clinical specimens of MPM at the DNA, mRNA and protein levels. In order to analyze the possible mechanisms of TUSC2 expression modulation, we studied genomic copy number variation in the TUSC2 locus using Representational Oligonucleotide Microarray Analysis (ROMA) and investigated a possible link between asbestos exposure and this gene expression. To better understand the role of TUSC2 in MPM, we performed Affymetrix microarray profiling of MPM cells transfected with the TUSC2 transgene and correlated these data with previously produced microarray profiles of clinical specimens of MPM to identify clinically relevant TUSC2 targets.

## Results

### Expression of TUSC2 mRNA is down-regulated in a large proportion of MPM tumors

**Figure 1:**
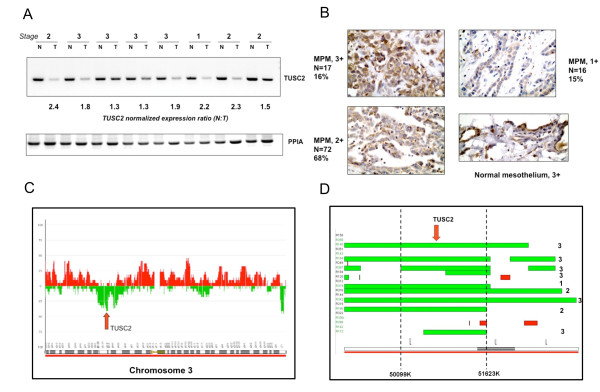
**Down-regulation of TUSC2 expression in mesothelioma**. **A**. TUSC2 mRNA levels are decreased in the majority of mesotheliomas. Semi-quantitative RT-PCR analysis of RNA isolated from matched normal and tumor specimens derived from mesothelioma patients. Upper panel (reproduced from [23]) displays RT-PCR products obtained with TUSC2-specific primers; bottom panel represents a loading control - RT-PCR products with the primers specific for PPIA, an invariantly expressed gene. N- normal tissue, T- tumor. Numbers at top represent disease stages, numbers at bottom show normalized fold change. **B**. Representative examples of TUSC2 immunohistochemical staining of mesothelioma and normal peritoneum. Intensity levels of TUSC2 staining designated as, 3+, 2+ and 1+ are illustrated by representative images.** C**. Loss of the 3p21.3 chromosomal area in mesothelioma tumor specimens as identified by ROMA. Synoptic map of DNA copy number variation in chromosome 3 in mesotheliomas (FDR = 0.09). Arrow shows the site of recurrent loss of TUSC2-containing 3p21.3 region in mesothelioma patients (n = 22). **D**. Detailed view of the 3p21.3 region losses in different mesothelioma patients and identification of common deletion area. Green boxes represent deletions detected in patients diagnosed with different stages of mesothelioma while red sector show gains. Coded patient IDs are shown in the left and disease stages in the right.

To find out whether TUSC2 mRNA expression is modulated in MPM we analyzed its expression in tumors of different histology, stage, and clinical behavior by semi-quantitative RT-PCR. For this study, we used a set of 30 matched specimens of unaffected peritoneum and MPM tumors. We found that TUSC2 is down-regulated in ~83% (25 out of 30) of MPMs as compared to matched normal mesothelium (see representative panel in Fig. 1A). We demonstrated as well that TUSC2 expression is independent of the disease histology and stage (data not shown) suggesting that TUSC2 down-regulation may be an early event in mesothelioma development.

### Down-regulation of the TUSC2 protein in MPM specimens

Our set of MPM specimens used for immunohistochemical studies included epithelial, sarcomatoid, and mixed (biphasic) histological types. We used specific anti-TUSC2 antibodies successfully tested previously on a set of human lung cancer samples [19]. We pooled the sarcomatoid and biphasic tumors in one group based on their common sarcomatoid component (Table 1). The TUSC2 protein was localized in the cytoplasm of tumor cells. Overall, ~88% (53 out of 60) of epithelial and ~80% (36 out of 45) of biphasic/sarcomatoid MPMs showed low or intermediate levels of TUSC2 expression (S1). We noted that the difference between the epithelial and sarcomatoid/biphasic types was not statistically significant (*p *= 0.09). Overall, MPM specimens showed lower scores and levels of TUSC2 expression as compared to normal mesothelium that was scored at a 280-300 intensity level (Fig. 1B). Specifically, most MPM tumors including stage I (89 out of 105, ~85%) had a score of < 220, which was never associated with normal mesothelial tissue pointing to down-regulation of the TUSC2 protein in MPM. As above, no correlation with the stage was observed.

**Table 1:** Immunohistochemical study of TUSC2 expression in MPMs.

**Histology of tumors**	**No. of samples**	**TUSC2 score,** **mean (SD)**	**Low (0-100),** **n (%)**	**Intermediate (101-220)** **n (%)**	**High (221-300)** **n (%)**
Epithelial	60	157.3 (66)	11 (18)	42 (70)	7 (12)

Biphasic+sarcomatoid	45	172.7 (56)	6 (13)	30(67)	9 (20)

### The TUSC2 locus is frequently deleted in MPM tumors

Decreased TUSC2 expression in ~83% of MPMs prompted us to seek possible mechanism(s) of its down-regulation. DNA copy number alteration (CNA) data generated previously by ROMA on 22 MPM specimens compared with normal human DNA isolated from fibroblasts [23] were processed using the ACE (analysis of copy errors) algorithm available from CGH-Explorer [24], and plotted across chromosome 3 (Fig. 1C). We found that a ~1.5-6 Mb TUSC2-containing 3p21.3 region was under-represented in 8 out of 22 samples analyzed (~36%) and identified the boundaries of the characterized deletions in individual patients (Fig. 1D). Analysis of different tumor stages implied that the 3p21.3 deletion in MM is a frequent event in stages 2 and 3 but could also be detected as early as stage 1 (one out of five stage 1 patients studied showed the deletion).

### TUSC2 expression is decreased upon asbestos exposure

**Figure 2:**
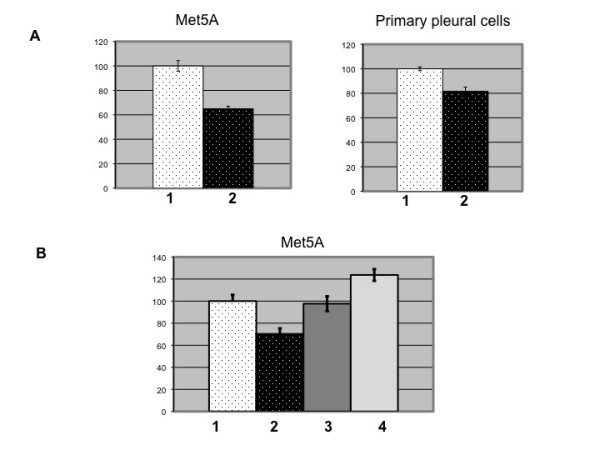
**TUSC2 expression is regulated by asbestos and reactive oxygen species**. **A**. TUSC2 is down-regulated by asbestos in mesothelial cell line Met5A and healthy primary culture of mesothelial cells. 1 - non-treated cells; 2 - cells treated with 2.5 μg/cm^2 ^crocidolite asbestos. **B**. TUSC2 suppression by asbestos is restored upon pre-incubation with reactive oxygen species (ROS) scavengers. Pre-treatment of cells with 500 U/mL catalase (bar 3) or 250 U/mL superoxide dismutase, (SOD) (bar 4) for 30' prior to addition of asbestos to Met5A cells. Data were obtained in triplicates via semi-quantitative RT-PCR and are expressed in percentage change from control (non-treated cells).

While TUSC2 allelic loss documented in ~36% of MPM patients is an important observation, it is not sufficient to explain the fact that up to ~83% of analyzed MPMs showed a decrease in the TUSC2 mRNA level. Since MPM is an asbestos-induced malignancy of the mesothelial layer, we hypothesized that TUSC2 expression could be modulated in normal mesothelial cells by asbestos exposure. We treated Met5A and primary peritoneal mesothelial cells with asbestos and analyzed TUSC2 level by semi-quantitative RT-PCR. Invariably expressed PPIA was used as a loading control. We found that TUSC2 was down-regulated by asbestos (Fig. 2A, bar 2) in both types of mesothelial cells suggesting that one of the stimuli that may cause TUSC2 decrease in MPM is exposure to asbestos.

### ROS scavengers prevent asbestos-induced TUSC2 downregulation

Studies on animal models and cell cultures confirmed that asbestos fibers generate reactive oxygen species (ROS) [25]. Hence, we asked if the observed asbestos-dependent TUSC2 suppression was due to asbestos-generated ROS. Pretreatment of the cells with catalase (a specific H_2_O_2 _scavenger) or superoxide dismutase SOD (a specific superoxide anion O_2_^- ^scavenger) negated TUSC2 downregulation (Fig 2B, bars 3 and 4). The results of these experiments provide a link between asbestos exposure, asbestos-generated ROS, and modulation of TUSC2 expression in mesothelial cells.

### Transcriptional changes triggered by TUSC2 overexpression in mesothelioma cells

**Figure 3:**
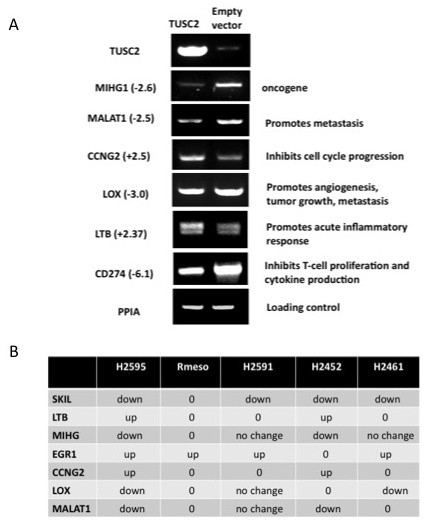
**RT-PCR validation of TUSC2-dependent changes in H2595 for selected genes**. **A**. Semi-quantitative RT-PCR analysis was done on RNA specimens used for Affymetrix profiling. Fold-estimation of up- (+) or down- (-)-regulation for each gene are shown in brackets. Invariably expressed PPIA gene served as a loading control. **B**. TUSC2 transcriptional targets were confirmed by semi-quantitative RT-PCR in four additional mesothelioma cell lines. TUSC2-dependent transcriptional change is shown as 'up' or 'down'; '0' indicates that PCR product was not detected.

We previously demonstrated on the mouse model that TUSC2 may affect mRNA levels of certain genes, such as IL15 [15]. To provide insight into the molecular basis of putative TUSC2 tumor suppressor activity in MPM and perform genome-wide analysis of TUSC2 transcriptional effects, we took advantage of human epithelioid MPM H2595 cells produced in our laboratory [26]. Since TUSC2 is commonly suppressed in MPM (see above) we used forced expression of TUSC2 to identify its targets. Analysis of these microarray data produced 228 probes corresponding to 195 genes modulated at least 2-fold by TUSC2 as compared to empty vector-transfected H2595 cells. We then performed biological profiling of TUSC2 targets using Xenobase software [27] for hypergeometric analysis of Gene Ontology annotation . These analyses identified groups of genes involved in organization of chromatin structure, cell differentiation, cell cycle, and immune system regulation (Table 2). Other statistically significant GO groups included 'intra-and inter-cellular signaling', 'molecular synthesis and metabolism', and 'homeostasis and secretion' categories. These findings suggest that TUSC2 activities may be related to vital cellular processes that are commonly affected during malignant transformation. Using NCBI annotation we then identified among the TUSC2 targets multiple genes previously associated with tumorigenesis (Table 3). Consistent with the TUSC2 tumor suppressor role, 21 genes with anti-tumorigenic properties including BCL211, FOXN3, GAS1, and NME5 were up-regulated by TUSC2. On the other hand, 20 pro-tumorigenic factors with JUN, ETS1, MALAT1, ATF3, and LOX among them were down-regulated. These microarray data were validated by semi-quantitative RT-PCR on several genes representing different functional groups (Fig. 3A).

**Table 2:** TUSC2 target genes grouped based on involvement in biological processes.

**Chromatin structure**	**P value**	**Cell cycle**	**P value**
nucleosome assembly	1.31E-06	cell growth and/or maintenance	0.0001
chromosome organization	1.97E-06	regulation of mitotic cell cycle	0.04
negative regulation of histone acetylation	0.007	cell cycle arrest	0.05
nucleosome disassembly	0.04	negative regulation of S phase	0.04
imprinting	0.04	negative regulation of cell proliferation	0.02
**Cell differentiation**	**P value**	**Immune system related activities**	**P value**
adipocyte differentiation	0.0008	complement activation	0.001
megakaryocyte differentiation	0.007	immune response	0.005
cell differentiation	0.01	antigen processing	0.007
**Intra-and inter-cellular signaling**	**P value**	innate immune response	0.02
cell-cell signaling	0.02	humoral immune response	0.04
cell surface receptor linked signal transduction	0.04	response to reactive oxygen species	0.01
regulation of G-pr. coupled receptor pr. signaling pathway	0.05	**Synthesis and metabolism**	**P value**
**Other proceses**	**P value**	glycolipid biosynthesis	0.02
viral assembly, maturation, and release	0.02	cAMP metabolism	0.02
lactation	0.04	valine metabolism	0.02
blood coagulation	0.04	regulation of hormone biosynthesis	0.03
transcription from Pol II promoter	0.04	nucleotide catabolism	0.03
**Homeostasis and secretion**	**P value**	proteoglycan metabolism	0.04
growth hormone secretion	0.02		
calcium ion homeostasis	0.03		
insulin secretion	0.04		

Hypergeometric P-values define probability of involvement of potential TUSC2 in listed biological processes. Only statistically significant (p ≤ 0.05) associations are shown.

**Table 3:** List of anti-tumorigenic and pro-tumorigenic molecules modulated by introduction of TUSC2 in H2595 MPM cell line (expression array data).

**Anti-tumorigenic molecules** **up-regulated by TUSC2**	**Fold** **change**	**Pro-tumorigenic molecules** **suppressed by TUSC2**	**Fold change**
**ALDH6A1**, aldehyde dehydrogenase 6 family	+2.5	**ATF3**, activating transcription factor 3	-2.7

**APOL6**, apolipoprotein L, 6	+2.0	**CD24**, CD24 molecule	-3.1

**BCL2L11**, BCL2-like 11 (apoptosis facilitator)	+2.0	**CD274**, CD274 antigen; programmed cell death 1 ligand 1	-6.2

**CCDC98**, coiled-coil domain containing 98	+2.3	**CTGF**, connective tissue growth factor	-2.0

**CCNG2**, cyclin G2	+2.5	**EFNB2**, ephrin-B2	-2.0

**EGR1**, early growth response 1	+2.4	**GPR87**, G protein-coupled receptor 87	-2.3

**EXT1**, exostoses (multiple) 1	+2.1	**ELK4**, ETS-domain protein (SRF accessory protein 1)	-2.2

**FBXO4**, F-box protein 4	+2.1	**JUN**, jun oncogene	-2.5

**FOXN3**, forkhead box N3	+2.6	**IL11**, interleukin 11	-3.9

**GAS1**, growth arrest-specific 1	+2.1	**LOX**, lysyl oxidase	-3.0

**MCC**, mutated in colorectal cancers	+2.1	**MALAT1**, metastasis associated lung adenocarcinoma transcript 1	-2.5

**MLL5**, myeloid/lymphoid or mixed-lineage leukemia 5	+2.5	**MIHG1**, microRNA host gene	-2.6

**NME5**, non-metastatic cells 5	+2.4	**PLAU**, plasminogen activator, urokinase	-3.2

**RARRES1**, retinoic acid receptor responder (tazarotene induced) 1	+4.1	**PLAUR**, plasminogen activator, urokinase receptor	-2.1

**RARRES3**, retinoic acid receptor responder (tazarotene induced) 3	+2.8	**RGS4**, regulator of G-protein signaling 4	-4.3

**SELENBP1**, selenium binding protein 1	+2.1	**SET**, SET translocation (myeloid leukemia-associated)	-2.2

**SIDT2**, SID1 transmembrane family, member 2	+2.3	**SKIL**, SKI-like oncogene	-2.3

**SAMD9L**, sterile alpha motif domain containing 9-like	+2.0	**THBS1**, thrombospondin 1	-2.5

**TNS1**, tensin 1	+2.5	**B4GALT1**, UDP-Gal:betaGlcNAc beta 1,4-galactosyltransferase	-2.5

**WFDC2**, WAP four-disulfide core domain 2	+3.5	**ETS1**, v-ets erythroblastosis virus E26 oncogene	-2.3

**WSB1**, WD repeat and SOCS box-containing 1	+2.0		

To validate these TUSC2-dependent transcriptional changes we overexpressed TUSC2 in four additional MPM cell lines and performed RT-PCR evaluation of several potential TUSC2 targets. Despite high intra-individual variation typical for mesothelioma [24] we were able to confirm all seven gene-candidates in at least two cell lines (Fig. 3B). Moreover, expression of SKIL, a negative regulator of TGF-beta signaling [28], and EGR1, a potent transcription factor with a dual role in tumorigenesis [29], was modulated upon TUSC2 introduction in 4 out of 5 tested MPM cell lines.

**Figure 4:**
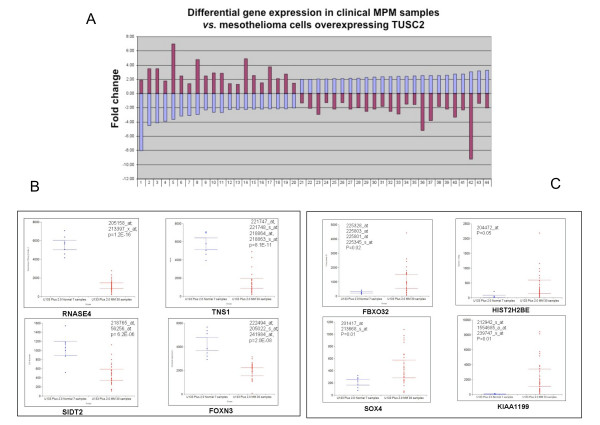
**Opposite directions of TUSC2 targets modulation in HP2595/TUSC2 versus mesothelioma tumors**. **A**. Graph shows fold change expression of 44 genes in mesotheliomas as opposed to MPM cells overexpressing TUSC2. Expression changes in mesothelioma are shown with red bars while in HP2595 cell line with blue bars. Only statistically significant modulation in mesothelioma as compared to normal peritoneum (p < 0.05) is shown. Gene names that correspond to numbers in the bottom are provided in Table 4. **B**. Representative plots for genes down-regulated in mesothelioma with high p-value, but upregulated by TUSC2. **C**. Representative plots for genes upregulated in mesothelioma with high p-value, but down regulated by TUSC2. Each plot shows Affymetrix IDs for each gene probes and respective p-value.

To assess clinical significance of newly identified TUSC2 targets we used our previously produced MPM expression array data set (30 MPM and 7 control specimens) and found that more then 21 genes down-regulated by TUSC2 in H2595 were up-regulated in clinical specimens of MPM and, vise versa, 24 genes up-regulated by TUSC2 in H2595 were suppressed in MPM with significant *p*-values (Fig. 4A and Table 4). The plots that illustrate expression of some of these genes in MPM are shown in Fig. 4B and 4C. Overall, these data imply that up to ~23% of cancer-associated TUSC2 targets may contribute to MPM progression and serve in future as novel therapeutic targets.

**Table 4:** Differentially expressed genes common for TUSC2-expressing MPM cells and clinical tumor specimens.

	**Fold change and direction in TUSC2-transfected MPM cells versus control**	**Fold change and direction in MPM versus healthy peritoneum**	**Gene product name**	**Gene ID**
1	-8.04	1.90	ribosomal protein S11	RPS11
2	-4.51	3.50	histone cluster 1, H2ae	HIST1H2AE
3	-4.10	3.50	regulator of G-protein signaling 4	RGS4
4	-3.90	1.80	parathyroid hormone-like hormone	PTHLH
5	-3.64	7.00	histone cluster 1, H2bg	HIST1H2BG
6	-3.14	2.50	CD24 molecule	CD24
7	-3.08	1.40	proteasome (macropain) subunit, beta type, 4	PSMB4
8	-2.92	4.80	lysyl oxidase	LOX
9	-2.31	2.50	protein phosphatase 1, regulatory subunit 15A	PPP1R15A
10	-2.59	2.90	histone cluster 1, H4 h	HIST1H4H
11	-2.67	2.87	activating transcription factor 3	ATF3
12	-2.26	1.40	SET translocation (myeloid leukemia-associated)	SET
13	-2.22	1.30	histone cluster 2, H2be	HIST2H2BE
14	-2.22	4.87	hypothetical protein LOC284801	LOC284801
15	-2.21	2.51	transmembrane protein 158	TMEM158
16	-2.18	1.50	histone cluster 2, H2aa3	HIST2H2AA3
17	-2.14	3.75	F-box protein 32	FBXO32
18	-2.12	2.10	histone cluster 1, H3d	HIST1H3D
19	-2.12	2.73	SRY (sex determining region Y)-box 4	SOX4
20	-2.04	1.48	fem-1 homolog b (C. elegans)	FEM1B
21	2.02	-1.35	BCL2-like 11 (apoptosis facilitator)	BCL2L11
22	2.02	-2.10	WD repeat and SOCS box-containing 1	WSB1
23	2.03	-2.96	chromosome 1 open reading frame 21	C1orf21
24	2.04	-1.30	muscleblind-like (Drosophila)	MBNL1
25	2.10	-2.20	selenium binding protein 1	SELENBP1
26	2.12	-1.28	butyrophilin, subfamily 3, member A1	BTN3A1
27	2.13	-2.20	AF4/FMR2 family, member 4	AFF4
28	2.18	-1.98	transmembrane protein 47	TMEM47
29	2.24	-2.52	superoxide dismutase 2, mitochondrial	SOD2
30	2.30	-2.20	SID1 transmembrane family, member 2	SIDT2
31	2.35	-1.82	transmembrane and coiled-coil domain family 1	TMCC1
32	2.36	-2.52	early growth response 1	EGR1
33	2.40	-2.90	aldehyde dehydrogenase 6 family, member A1	ALDH6A1
34	2.44	-1.50	cyclin G2	CCNG2
35	2.50	-1.54	cell division cycle 2-like 6 (CDK8-like)	CDC2L6
36	2.52	-5.20	ribonuclease, RNase A family, 4	RNASE4
37	2.53	-3.80	tensin 1	TNS1
38	2.54	-1.80	myeloid/lymphoid or mixed-lineage leukemia 5	MLL5
39	2.57	-2.20	forkhead box N3	FOXN3
40	2.75	-3.32	zinc finger E-box binding homeobox 1	ZEB1
41	2.76	-2.30	protocadherin 17	PCDH17
42	3.06	-9.20	cytochrome P450, family 4, subfamily F	CYP4F3
43	3.18	-1.36	complement component 3	C3
44	3.30	-2.03	AT rich interactive domain 5B (MRF1-like)	ARID5B

Comparison between expression array data sets reveals 44 concordantly regulated gene

Our previous data produced on Tusc2 KO mice suggested that TUSC2 is an important regulatory component of immune system, which is able to stimulate IL-15 expression as well as maturation of NK cells and lymphocytes [15]. In agreement with the mouse KO data, one of the multiple targets modulated by TUSC2 in MPM cells was IL15 that was 2-fold up-regulated by FUS1 introduction. Other genes previously associated with the immune system included IL11 (4-fold down-regulated by TUSC2), CD24 and CD274 (Table 3), CD54/ICAM1 (2-fold up-regulated by FUS1) as well as ~2-3-fold up-regulated complement components C1s, CFB, and C3 (Table 4). To further explore the immune link of TUSC2 we took advantage of the BioGPS portal developed by the Novartis Research Institute . This analysis identified among TUSC2 targets 46 human genes that show exceptionally high levels of expression in immune cells (Table 5). Noteworthy, expression of most of them (marked with asterisks) was specific to immune tissues and cells and seemed to be dependent on the activation status and cell type. At this time, the function of many of these genes is not yet known. Overall, our results imply that TUSC2 plays critical roles in cell cycle progression, cell differentiation, and immune system regulation, and that its downstream targets may provide important insights previously unknown molecular mechanisms and pathways involved in cellular defense against cancer.

**Table 5:** TUSC2 modulates 47 genes highly expressed in immune cells.

	**Gene name**	**TUSC2 effect**	**Expression** **Profile**
**1**	ADD3	UP	III

**2**	AHSA2	UP	III

**3**	BTN3A1*	UP	I

**4**	BTN3A2*	UP	I

**5**	BTN3A3*	UP	I

**6**	C7orf29*	UP	I

**7**	CAST*	DOWN	I

**8**	CCDC88A*	DOWN	I

**9**	CCNG2	UP	III

**10**	CD274*	DOWN	I

**11**	CXCL16	UP	II

**12**	DENND1B*	UP	I

**13**	DOCK11*	UP	I

**14**	EGR1	UP	II

**15**	ETS1*	DOWN	I

**16**	FOXN3	UP	III

**17**	GADD45A	DOWN	III

**18**	GADD45B	DOWN	III

**19**	HCP5*	UP	I

**20**	HIST1H2AE	DOWN	II

**21**	HIST1H2BG*	DOWN	I

**22**	HIST1H3D*	DOWN	I

**23**	HPSE*	UP	I

**24**	ICAM1/CD54	UP	II

**25**	IL15*	UP	I

**26**	JUN	DOWN	III

**27**	LTB*	UP	I

**28**	MALAT1*	DOWN	I

**29**	MBNL*	UP	I

**30**	METTL4*	UP	I

**31**	MIB1	UP	II

**32**	MLL5	UP	III

**33**	NT5E	DOWN	II

**34**	PARP9*	UP	I

**35**	PLAUR	DOWN	III

**36**	PPP1R15A*	DOWN	II

**37**	PSMB9*	UP	I

**37**	RGS2*	DOWN	I

**39**	SELENBP1	UP	II

**40**	SNX5	DOWN	III

**41**	SOX4*	DOWN	I

**42**	TncRNA	DOWN	II

**43**	TNRC6B	UP	III

**44**	TNS1*	UP	I

**45**	UBE2L6*	UP	I

**46**	ZNF224*	UP	I

**47**	ZNF652	UP	II

Profile I - genes expressed exclusively in immune cells; Profile II- genes expressed mainly in immune cells; Profile III - widely expressed genes that show highest expression levels in the immune compartments. Expression profiling was performed via .

### TUSC2 similarity to the IRF7 DNA-binding domain

**Figure 5:**
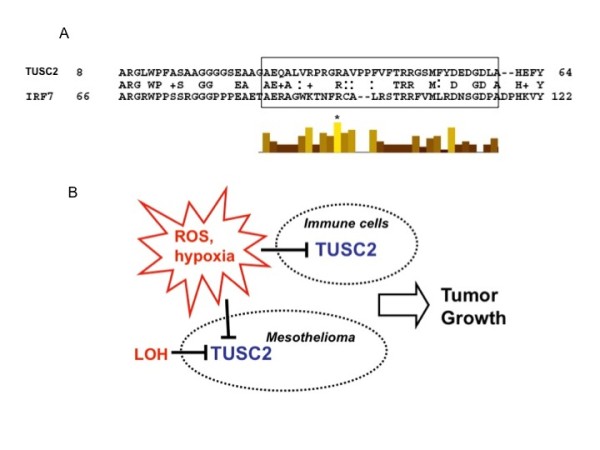
**Molecular and biological properties of TUSC2**. **A**. Similarity with the interferon regulatory factor IRF7 suggests DNA-binding property for TUSC2. N-terminus of TUSC2 shows ~40% identity with the IRF7 segment that contains a DNA-binding site (framed). Histogram shows positions conserved in the IRF protein family. The arginine residue (R) conserved throughout the IRF protein family is marked with asterisk. **B**. Different mechanisms of TUSC2 down-regulation in tumor and immune cells. Modulation of TUSC2 in tumor and host immune cellular compartments may contribute to tumor growth via blocking TUSC2 tumor suppressive or immune-regulatory properties.

Prominent transcriptional effects triggered by TUSC2 over-expression in MPM suggested that it may function as an essential transcriptional regulator. In this study, we therefore analyzed in more detail the similarity between the TUSC2 and IRF7 proteins that we reported previously [15]. As shown in Fig. 5A, the N-terminus of TUSC2 displays similarity with the DNA-binding domain of IRF7. Multiple alignment performed within the IRF family using TCoffee  helped us to identify evolutionary conserved amino acid residues in this domain. Noteworthy, most of the TUSC2 amino acid residues in this area follow the functional conservation pattern including the only invariable residue Arg96 (shown with asterisk in Fig. 5A). The similarity with the IRF7 DNA-binding domain is consistent with the observed TUSC2 effects on immune genes. In addition, our *in silico *analysis confirmed the presence of IRF and NF-kB-binding sites in the promoters of newly identified TUSC2 targets (data not shown). Mutational analysis of the putative DNA-binding TUSC2 domain is required, however, to answer if TUSC2 is a *bone fide *transcriptional regulator.

## Discussion

TUSC2 resides in the critical ~120 kb region that is often deleted in lung, breast, head and neck, renal, and other cancers [10]. While studies on lung cancer cell lines revealed a limited (~4%) frequency of mutations in the TUSC2 gene [10,16] and infrequent methylation of its promoter [16], large-scale analysis of TUSC2 expression in lung cancers and in bronchial squamous metaplastic and dysplastic lesions have revealed significantly lower levels of TUSC2 as compared to normal hyperplastic epithelia [30]. These data suggest that TUSC2 down-regulation may play an important role in early pathogenesis of lung cancer.

Targeted disruption of the Tusc2 gene in mice that our group performed previously resulted in a complex immunological autoimmune phenotype with an inflammatory background and recurrent tumorigenesis at the sites of chronic inflammation [15]. Based on these data, we hypothesized that TUSC2 down-regulation may contribute to the development of human cancers associated with chronic inflammation. One such cancer is mesothelioma, an aggressive and mostly lethal malignancy linked with asbestos exposure. Our analysis of the TUSC2 mRNA and protein levels in MPM as compared to normal peritoneum confirmed the decrease in TUSC2 level in ~84% of tumors irrespective of stage and histological type. This observation suggests a fundamental importance of TUSC2 downregulation for MPM origin and progression. Incomplete TUSC2 down-regulation shown in our study is consistent with the haploinsufficient effect of the gene observed in our mouse KO model. Indeed, mice hetero- or homozygous on the Tusc2 deletion shared similar immunological and tumorigenic phenotypes at comparable frequencies. These data suggest that even partial loss of the TUSC2 dosage is sufficient to trigger pathological inflammation and increase susceptibility to cancer [15].

We considered several possible scenarios of TUSC2 inactivation in MPM. Since importance of LOH in the TUSC2 chromosomal area had been demonstrated for multiple cancers [10,16], we performed analysis of DNA copy number variation in the 3p21.3 region in MPM tumors. We found that deletion of the TUSC2 locus is a recurrent event in MPMs (~36%) substantiating the idea of LOH as a principal and clinically relevant mechanism of TUSC2 inactivation in MPM.

We then hypothesized that down-regulation of TUSC2 expression may be also triggered by carcinogenic stimuli. Since the principal role of asbestos in MPM has been supported by many independent studies we investigated if TUSC2 expression can be affected by exposure to asbestos. We found that the TUSC2 expression level was decreased in both types of tested mesothelial cells upon 24 h of asbestos exposure, further corroborating the link between TUSC2 and MPM. Moreover, we showed that a short pre-treatment of cultured mesothelial cells with ROS scavengers completely abrogated the asbestos effect. This observation suggests that the suppressive effect of asbestos on TUSC2 expression is mediated via ROS. Indeed, generation of ROS by asbestos was documented in many studies [25]. A yet another source of ROS that may keep the TUSC2 level down in normal and malignant tissues are inflammatory cells attracted to the site of asbestos lodging [31]. We previously showed that TUSC2 is expressed in T- and B-cells and is involved in the Natural Killer (NK) cells maturation and function [15]. Therefore, down-regulation of TUSC2 in NK cells and other tumor-infiltrating lymphocytes by ROS may help cancer cells to escape immune suppression. Finally, since hypoxia is one of the most common physiological conditions during tumor growth we sought evidence of TUSC2 hypoxic link. As a result, we found in the NCBI Gene Expression Omnibus several experimental assays performed on different cell types that consistently showed TUSC2 mRNA suppression by hypoxia (see records GDS2759, GDS2018, GDS2760, and GDS2951 for FUS1/TUSC2 at ). Based on these data and the dual role of TUSC2 as an immune regulator and tumor suppressor it may be suggested that TUSC2 suppression during tumor growth may be caused my multiple factors and in different cells, namely immune cells present at the site of inflammatory tumor and cancer cells where the effect of LOH is further aggravated by ROS and hypoxia (Fig. 5B).

The role of TUSC2 as a tumor suppressor in lung cancer is widely accepted [16-18]. TUSC2 delivered in lipid-based nanoparticles drastically reduced the number and size of human non-small cell lung cancer tumors in mice [19]. The TUSC2 nanoparticles are being tested in a phase 1 safety and dose-escalation clinical trial at the M. D. Anderson Cancer Center for patients with advanced non-small cell lung cancer [32].

To provide insight into the pathways and genes affected by TUSC2 in MPM, we performed expression microarray profiling of mesothelioma cells transfected with TUSC2. As a result, we delineated several groups of genes under the TUSC2 control. Interestingly, genes involved in chromatin modulation, nucleosome assembly and histone acetylation demonstrated the most robust *p*-values (0.04 < p < 1.3E-06) indicating that TUSC2 transcriptional effects are most likely mediated via epigenetic modulation of chromatin function. In addition, forced expression of TUSC2 affected genes involved in cell cycle regulation, differentiation, and cell growth (0.01 < p < 0.0008) suggesting that TUSC2 possesses principal tumor suppressor properties. Finally, among newly identified TUSC2 targets we isolated more than 40 genes that may play important but not yet established roles in NK, dendritic cells, and T- and B-lymphocytes activity. Looking for molecular mediators that may be relevant to lupus-like autoimmune phenotype observed in our TUSC2 knockout model [15] we noticed that at least two putative TUSC2 targets, CD24 and CD274/PD-L1/B7-H1 that regulate immunogenic T and B cells, are linked with systemic autoimmune diseases such as SLE, MS, and others [33-38]. Based on many studies, overexpression of CD274 in common cancers helps tumor progression contributing to evasion from immune surveillance [39]. Our data suggest that TUSC2 is a potent suppressor of this key immunoreceptor (Table 3 and Fig. 3). Since blocking of CD274 is now actively pursued as a novel immunotherapy [40,41], we suggest that TUSC2 may also have important immunotherapeutic implications.

Our study on the TUSC2 role in mesothelioma underlines two modes of its biological activity: as a "classical" tumor suppressor capable of regulating vital cellular processes such as cell growth, differentiation, and death; and as an immune system regulator that affects multiple genes expressed in lymphocyte compartments. Characterization of a DNA-binding domain in the TUSC2 molecule makes it possible to further analyze its transcriptional activities through mutational analysis. The similarity between TUSC2 and IRF7 is especially important since this IFN-activated transcriptional factor is a principal component of the anti-viral response initiated in mitochondria [42,43]. Since TUSC2 is a mitochondrial protein, our findings support an important role for mitochondria in the immune defense against cancer and bring to light previously unrecognized molecular components of mitochondrial immunity.

## Conclusion

TUSC2 is a novel tumor suppressor from the critical 3p21.3 chromosomal region frequently deleted in multiple cancers that plays a critical role in lung cancer development. While clinical trial on restoration of TUSC2 activities in lung cancer patients is underway, its status and role in mesothelioma, an aggressive thoracic inflammatory cancer associated with exposure to asbestos, had never been established.

We demonstrated earlier that Tusc2-deficient mice display a complex immuno-inflammatory phenotype with a predisposition to cancer. The data presented here provide the first evidence that the TUSC2 protein plays an essential tumor suppressive role in asbestos-induced mesothelioma. This conclusion is supported by the decreased TUSC2 levels in the majority of MPM specimens and down-regulation of TUSC2 expression by asbestos-generated reactive oxygen species (ROS). On the molecular level, we associated TUSC2 with activation of multiple tumor-suppressor genes, down-regulation of oncogenes, and modulation of numerous immune genes. Overall, our study suggests that restoration of TUSC2 activities in MPM patients may have important therapeutic implications.

## Methods

### Mesothelioma specimens and RNA isolation

All patients gave informed consent for de-identified use of tissues under the Wayne State University IRB approved tissue consent document D1420: Collection of Serum and Tissue Samples for Patient's with Biopsy Proven or Suspected Malignant Disease. Immediately after surgical resection tissues were snap frozen and kept at -80°C until RNA isolation. For RT-PCR assessment of TUSC2 expression we used tumor/control matched specimens derived from thirty MPM patients. Control specimens were derived from unaffected peritoneum. RNA from these specimens was extracted using MirVana kit (Applied Biosystems, Foster City, CA) for subsequent TUSC2 RT-PCR analyses.

### TMA slides

Immunohistochemical analysis was performed using two sources of multi-tissue pleural mesothelioma arrays (TMA). Three TMAs were made using MPM samples obtained from patients at Karmanos Cancer Institute (Detroit, MI). They contained a total of 30 cases represented by 2-mm cores of resected tumors and adjacent mesothelial tissues. A validating set of four TMA slides containing 75 MPM cases from the MD Anderson Cancer Center (Houston, TX) were constructed using triplicate 1 mm diameter cores from each tumor. All patients gave informed consent for de-identified use of tissues. Both studies were approved by the Wayne State University and The University of Texas IRB Institutional Review Boards.

### Immunohistochemical staining

Rabbit anti-TUSC2 polyclonal antibodies used for immunohistochemical staining were raised against a synthetic oligopeptide derived from NH_2_-terminal amino acid sequence (NH_2_-GASGSKARGLWPFAAC) [16]. Formalin-fixed and paraffin-embedded tissue histology sections (5 μm thick) were deparaffinized, hydrated, and heated in a steamer for 10 min. with 10 mmol/L of sodium citrate (pH 6.0) for antigen retrieval. Peroxide blocking was done with 3% H_2_O_2 _in methanol at room temperature for 15 min, followed by 10% bovine serum albumin in TBS-t for 30 min. The slides were incubated with primary antibody at a 1:400 dilution for 65 min at room temperature. After washing with PBS, incubation with biotin-labeled secondary antibody for 30 min followed. Finally, the samples were incubated with a 1:40 solution of streptavidin-peroxidase for 30 min. The staining was then developed with 0.05% 3',3-diaminobenzidine tetrahydrochloride prepared in 0.05 mol/L of Tris buffer at pH 7.6 containing 0.024% H_2_O_2 _and then counterstained with hematoxylin. Normal mesothelial tissue was used as a positive control. For a negative control, we used the same specimens used for the positive controls, replacing the primary antibody with PBS.

### Subcellular and tissue distribution of the TUSC2 protein

TUSC2 immunostaining was detected in the cytoplasm of mesothelial and tumor cells (Fig. 1). Immunohistochemical expression was quantified in a blinded fashion by two independent pathologists (LP and DN) using a four-value intensity score (0, 1+, 2+, and 3+) and the percentage of the reactivity. A consensus value on both intensity and reactivity was reached by the two independent observers. A final consensual score was obtained by multiplying both intensity and extent of reactivity values (range, 0-300), and four levels of staining were calculated based on that score: (*a*) negative (score 0), (*b*) low (score ≤ 100), (*c*) intermediate (score > 100 to ≤ 220), and (*d*) and high (score > 220) expressions.

### ROMA and data processing

Since FUS1 has been previously associated with the 3p21.3 deletion in lung and other cancers [10], we used our previously produced Representational Oligonucleotide Microarray Analysis (ROMA) data [23] for assessment of this association in MPM specimens. ROMA, as compared to other array comparative genomic hybridization (aCGH) technologies, is better suited for translational research due to its higher sensitivity [44]. The arrays used for the ROMA analyses and data processing were described previously [44]. Briefly, for ROMA analysis, 27 snap-frozen specimens (22 tumor and 5 normal) derived from 22 patients with MPM were used to produce 27 DNA samples. 4 out of 5 normal samples were tumor-matched. DNA from snap-frozen tissues was isolated with QIAamp mini-kit (Qiagen, Valencia, CA) and assayed for concentration and quality using Victor3 microplate reader (PerkinElmer, Waltham, MA). Matching detailed clinical information for these specimens was available.

### Cell lines

SV40-transfected mesothelial cell line Met5A, primary pleural mesothelial cultured cells and H2595 epithelial MPM cells lines were grown in 1× DMEM supplemented with sodium pyruvate, high glucose and 10% fetal bovine serum (FBS) (Invitrogen, Carlsbad, CA). The H2595 cell line was established in our laboratory from a surgical specimen obtained by one of the authors (HP) [26].

### Asbestos and reactive oxygen species (ROS) experiments

We treated Met5A and primary pleural mesothelial cells with 2.5 μg/cm^2 ^of crocidolite asbestos for 24 hours. Pretreatment of the cells with 500 U/ml catalase (a specific H_2_O_2 _scavenger) or 250 U/ml superoxide dismutase SOD (a specific superoxide anion O_2_^- ^scavenger) was done for 30 min. prior to addition of asbestos. Both experiments were performed in triplicates and repeated twice.

### TUSC2 overexpressing constructs and transfection

The open reading frame (ORF) for forced expression of human TUSC2 was obtained by RT-PCR on human RNA. The amplified product was cloned in a polylinker-containing vector, excised with EcoRI and BamI, and subcloned into pCMV2/FLAG (Sigma-Aldrich, St. Louis, MO). Genes integrated in this vector are controlled by the CMV promoter. Sequence fidelity and accurate reading frame were verified by DNA sequencing. All cells in the study were transfected using Lipofectamin^2000 ^(Invitrogen, Carlsbad, CA) according to the manufacturer's protocol.

### Gene expression profiling and Xenobase analysis

Total RNA from TUSC2-transfected and vector-transfected MPM H2595 cell line were isolated with Qiagen RNeasy kit, analyzed for quality using an Agilent Bioanalyzer and hybridized with the Affymetrix U133 2.0 Plus microarray. Gene expression data were analyzed using Xenobase, a databank and software designed for translational studies in the Van Andel Research Institute (Grand Rapids, MI, [27]). Along with the *in vitro *cell line microarray data, we included into the analysis an Affymetrix U133 2.0 Plus data set previously obtained by us on thirty other MPM tumor specimens compared with normal peritoneum (*n *= 7) [23]. Profiling of Affymetrix data to identify statistically significant biological groups of genes was done using Xenobase software for hypergeometric analysis of gene annotations provided by the Gene Ontology Program .

### RT-PCR analysis

Reverse transcription - polymerase chain reaction (RT-PCR) was done using Super Script One-Step RT-PCR kit (Invitrogen, Carlsbad, CA). Primers for RT-PCR assessment of gene expression were designed as described [45]. Conditions for the reaction were as follows: 50°C - 30 min., 35 cycles of (94°C - 15 sec, 56°C - 15 sec, 72°C - 1 min.), 72°C - 5 min. Oligonucleotide sequences for RT-PCR were designed as follows: *LTB*: 5'-ATCACTGTCCTGGCTGTGCT-3', 5'-GCAGCACTGAAGCTTTCCAT-3'; *FUS1 (TUSC2)*: 5'-AACTCCCAGGCTCAATCAAG-3' -, 5'-CAGACTCTGCCACGACATC-3'; *MALAT1*: 5'-GAAGGTCTGAAGCTCATACC-3', 5'-CTTTCCATTACGCAACTGAG-3'; *MIHG1*: 5'-ATGTTTTGCCACGTGGATGT-3', 5'-GGCAATCATAACCAACCATC-3'; CCNG2: 5'-TTGTTGAACGTCTACCTGGA-3', 5'-CACTCTTGATCACTGGGAG-3'; LOX: 5'-GAGAAGTTCCTGCGCTCAGT-3', 5'-ACCAGGCACTGATTTATCCA-3'. RT-PCR with primers to invariantly expressed PPIA (5'-TCTGAGCACTGGAGAGAAAGG-3', 5'-GGAAAACATGGAACCCAAAGG-3') was used as a loading control. Band intensities were measured using Kodak 4000 Image Station. Repeated experiments showed consistency of our RT-PCR analysis.

### Statistical evaluation

Results are expressed as percentages or medians and ranges. Statistical analysis included two sided Student's *t*-test reported as mean standard error. Statistical significance was considered at *p *< 0.05.

## List of Abbreviations

MM: Malignant Pleural Mesothelioma; ROS: Reactive Oxygen Species; TMA: Tissue MicroArray; ROMA: Representational Oligonucleotide Microarray Analysis; CAN: Copy Number Alteration; ACE: Analysis of Copy Errors.

## Competing interests

The authors declare that they have no competing interests.

## Authors' contributions

AI designed the study, participated in its coordination, carried out the molecular studies on asbestos and ROS effects on TUSC2 expression, was involved in the analysis of microarray data and their molecular confirmation, processed all the data and wrote the manuscript, SI carried out mesothelioma genomic and transcriptomic database analyses, was involved in molecular experimentations and helped to draft two chapters of the manuscript, LP performed the analysis and scoring of immunostained mesothelioma tissues, DN performed the analysis and scoring of immunostained mesothelioma tissues independently of LP, AT performed an initial analysis of stained mesothelioma samples, IW coordinated the mesothelioma tissue acquisition and staining from MD Anderson Cancer Center site and participated in preparation of Methods section and discussion of the results, ZL performed the analysis of microarray data, JR participated in discussion of the data and the manuscript, HP conceived of the study, and participated in its design and coordination and was involved in tissue acquisition and collection of patients' clinical data. All authors read and approved the final manuscript.
